# Closed‐Loop Recycling of Wearable Electronic Textiles

**DOI:** 10.1002/smll.202407207

**Published:** 2024-10-02

**Authors:** Marzia Dulal, Shaila Afroj, Md Rashedul Islam, Minglonghai Zhang, Yadie Yang, Hong Hu, Kostya S. Novoselov, Nazmul Karim

**Affiliations:** ^1^ Centre for Print Research The University of the West of England Bristol BS16 1QY UK; ^2^ Department of Textile Engineering Management Bangladesh University of Textiles (BUTEX) Tejgaon Industrial Area Dhaka 1208 Bangladesh; ^3^ Faculty of Environment, Science and Economy Department of Engineering University of Exeter Exeter EX4 4QF UK; ^4^ Department of Wet Process Engineering Bangladesh University of Textiles (BUTEX) Tejgaon Industrial Area Dhaka 1208 Bangladesh; ^5^ School of Fashion and Textiles the Hong Kong Polytechnic University Kowloon 999077 Hong Kong; ^6^ Institute for Functional Intelligent Materials Department of Materials Science and Engineering National University of Singapore Singapore 117575 Singapore; ^7^ Nottingham School of Art and Design Nottingham Trent University Shakespeare Street Nottingham NG1 4GG UK; ^8^ Department of Fashion and Textiles University of Southampton Southampton SO23 8DL UK

**Keywords:** closed‐loop recycling, e‐textiles, graphene, sustainability, wearable

## Abstract

Wearable electronic textiles (e‐textiles) are transforming personalized healthcare through innovative applications. However, integrating electronics into textiles for e‐textile manufacturing exacerbates the rapidly growing issues of electronic waste (e‐waste) and textile recycling due to the complicated recycling and disposal processes needed for mixed materials, including textile fibers, electronic materials, and components. Here, first closed‐loop recycling for wearable e‐textiles is reported by incorporating the thermal‐pyrolysis of graphene‐based e‐textiles to convert them into graphene‐like electrically conductive recycled powders. A scalable pad‐dry coating technique is then used to reproduce graphene‐based wearable e‐textiles and demonstrate their potential healthcare applications as wearable electrodes for capturing electrocardiogram (ECG) signals and temperature sensors. Additionally, recycled graphene‐based textile supercapacitor highlights their potential as sustainable energy storage devices, maintaining notable durability and retaining ≈94% capacitance after 1000 cycles with an areal capacitance of 4.92 mF cm⁻^2^. Such sustainable closed‐loop recycling of e‐textiles showcases the potential for their repurposing into multifunctional applications, promoting a circular approach that potentially prevents negative environmental impact and reduces landfill disposal.

## Graphical abstract

A closed‐loop recycling for wearable e‐textiles is developed by incorporating the thermal‐pyrolysis of graphene‐based e‐textiles to convert them into graphene‐like electrically conductive recycled powders. A scalable pad‐dry coating technique is then used to reproduce graphene‐based wearable e‐textiles and demonstrate their potential wearable energy storage and healthcare applications for capturing electrocardiogram (ECG) signals and temperature sensors.

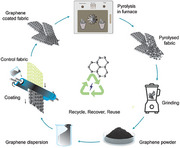

## 1 Introduction

Electronic waste, also known as e‐waste, is one of the fastest‐growing solid waste streams,^[^ ^1^ ^]^ millions of electrical and electronic devices are thrown away yearly due to failures or obsolescence.^[^ ^2^ ^]^ As e‐waste contains hazardous elements, improper management of this waste category can seriously affect the environment and public health. Additionally, a significant amount of energy and resources are used during the entire lifecycle of electronic equipment, from production to disposal. However, only ≈17% of global e‐waste is currently collected, sorted, and recycled,^[^ ^3^ ^]^ which poses huge sustainability challenges for e‐waste management. In addition to effective collection, sorting, and issues with the nonuniformity of the collected e‐waste, one of the major challenges in managing e‐waste is establishing sustainable and effective closed‐loop recycling processes.^[^ ^3^, ^4^ ^]^ The rapidly growing interest in developing wearable e‐textiles will exacerbate the e‐waste problem. Additionally, integrating electronics into textiles for e‐textile manufacturing further complicates the recycling and disposal processes due to the mix of materials—textile fibers, electronic materials, and electronic components—making them harder to separate and process efficiently.^[^ ^5^ ^]^

Wearable e‐textiles, with their ability to detect various stimuli and capture a wide range of signals using a single device, are incredibly valuable for personalized healthcare applications.^[^ ^6^, ^7^, ^8^, ^9^, ^10^, ^11^, ^12^, ^13^, ^14^ ^]^ The current limitations of e‐textiles, such as poor washability, instability, and subpar sensing capabilities, pose significant challenges to their widespread and practical use in areas like personal healthcare management, virtual gaming, sports, and beyond.^[^ ^15^, ^16^, ^17^ ^]^ Nevertheless, their broader acceptance is hindered by sustainability challenges as well such as inadequate material durability and eco‐friendliness, complex and lengthy production processes with significant carbon emission and toxic waste generation during manufacture, and difficulties in recycling or disposal at the end‐of‐life (EoL).^[^ ^18^, ^19^ ^]^ Textiles, which are the major component of e‐textiles, are considered the second largest polluter of the environment with an estimated ≈92 million tons of textile waste inundating landfills, even though 95% of textiles are entirely recyclable.^[^ ^20^ ^]^ The inefficiency in textile recycling exacerbates environmental degradation and highlights the pressing need for sustainable solutions. The integration of electronic materials into conventional textiles for e‐textile manufacturing adds a layer of complexity to their EoL processing. Additionally, these e‐textiles often contain non‐textile components like electronics, batteries, and interconnections, rendering disassembly a daunting task. E‐waste recycling technologies are being investigated as a potential solution to current e‐waste problems.^[^ ^21^, ^22^ ^]^ However, existing recycling strategies have mainly targeted e‐waste from traditional electronic devices, making them ineffective for recycling of wearable e‐textiles. While there have been initiatives to recycle flexible, soft and bioinspired electronics^[^ ^23^, ^24^, ^25^, ^26^ ^]^ through methods like substrate reuse,^[^ ^27^, ^28^ ^]^ conductive material recovery^[^ ^28^, ^29^ ^]^ (including silver nanowires^[^ ^30^, ^31^ ^]^ and liquid metals),^[^ ^27^, ^32^ ^]^ and carbon‐based component reclamation,^[^ ^33^, ^34^ ^]^ these approaches do not apply to the recycling or component separation of wearable e‐textiles. Therefore, there remains an unmet need for an effective and sustainable closed‐loop recycling technology for the rapidly growing wearable e‐textiles industry.

Here, the first closed‐loop recycling approach for smart wearable e‐textiles is reported. Our eco‐friendly approach includes 1) the use of sustainable materials including Tencel (also known as Lyocell) as textile fabrics, which are renewable, biodegradable and considered ecofriendly with added softness and comfort and graphene as conductive materials which were industrially produced by sustainable loop technology, involving low‐energy microwave plasma cracking process of biomethane waste to produce graphene; 2) a highly effective and resource‐efficient closed‐loop recycling of wearable e‐textiles via a pyrolysis process; and 3) upcycling of recycled materials for reuse as conductive graphene‐like materials for coating Tencel fabric and demonstrating their potential applications as multifunctional wearable devices including ECG, temperature sensor and supercapacitors. There are some considerable advantages to using this approach. Firstly, the process utilizes sustainable materials such as Tencel fabric, which is a cellulose‐based structure that acts as a carbon precursor for producing biochar or carbon‐based compounds after recycling. Secondly, thermal recycling via pyrolysis can break down these organic materials at higher temperatures without oxygen, producing biochar. Such a recycling process yields valuable benefits such as waste reduction, as it can convert our bio‐based wastes into useful material and retain carbon, which lowers greenhouse gas emissions by locking carbon in a stable form.^[^ ^35^, ^36^ ^]^ Also, by‐products produced from the pyrolysis of bio‐based materials are utilized as conductive material as an alternative solution to expensive or rare metals. Finally, this provides an opportunity to recover materials upcycle into new multifunctional, value‐added goods. By prioritizing waste prevention in sustainable design concepts for e‐textiles, these tools play a critical role in helping supply chain stakeholders make informed decisions, reduce environmental impact, and achieve sustainability goals.

## 2 System Overview: Closed‐Loop Recycling of Wearable E‐Textiles

**Figure 1:**
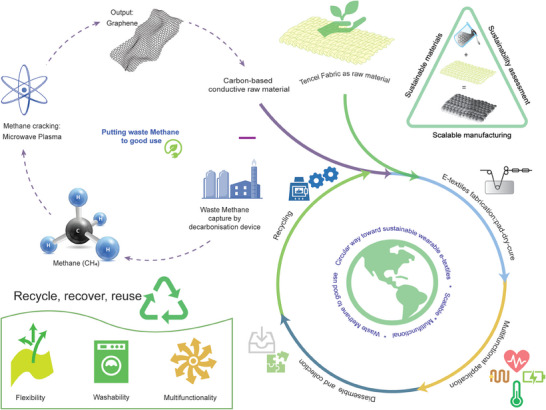
A comprehensive exploration of EoL options for wearable e‐textiles. The process involves repurposing waste methane‐derived graphene through scalable manufacturing, incorporating multifunctional applications, disassembling electrical components from textile electrodes, and analyzing EoL options. The sequence includes heat treatment by pyrolysis and mechanical recycling methods.

**Figure** **1** illustrates a comprehensive closed‐loop recycling process for wearable e‐textiles, which consists of both fabric and electronic materials. The approach is initiated with sustainable materials, such as sustainable Tencel fabric. Moreover, the electrically conductive graphene as supplied is industrially produced by a sustainable approach (Loop decarbonization device) which uses low‐energy microwave plasma cracking of biomethane waste and obviates the requirement for additives or catalysts. Such a process tackles methane emissions by converting the gas into graphene, reducing greenhouse gas emissions.^[^ ^37^ ^]^ Graphene can be extracted through the efficient channeling of released gas into methane cracking systems.

We then used a scalable method^[^ ^38^ ^]^ for graphene deposition on Tencel fabric using a laboratory‐scale pad‐dry‐cure machine, traditionally utilized for applying functional finishes to textiles. This method, capable of coating approximately 150 m fabric per min, was adapted to a smaller scale to demonstrate its feasibility for large‐scale e‐textile production. The process involved passing Tencel fabric through a concentrated graphene dispersion (100 g L^−1^), followed by the removal of excess liquid, resulting in an instant color change to black due to the graphene. After coating, the graphene was dried for 5 min at 80 °C to adhere it to the fabric; further layers might be added to increase functionality. Scanning electron microscope (SEM) images (Figure 3f) confirmed that graphene flakes were successfully deposited on fibers, resulting in a conductive fabric that is ideal for multifunctional uses of wearable e‐textiles as electrodes. Wearable e‐textiles thus produced were demonstrated for various potential applications, including textile electrodes for ECG monitoring, temperature sensors, and supercapacitors. Before being processed as feedstock for the recycling of wearable e‐textiles, the connecting wire and tapes, which were attached during device fabrication and performance testing, were disassembled from the textile electrodes.

**Figure 2:**
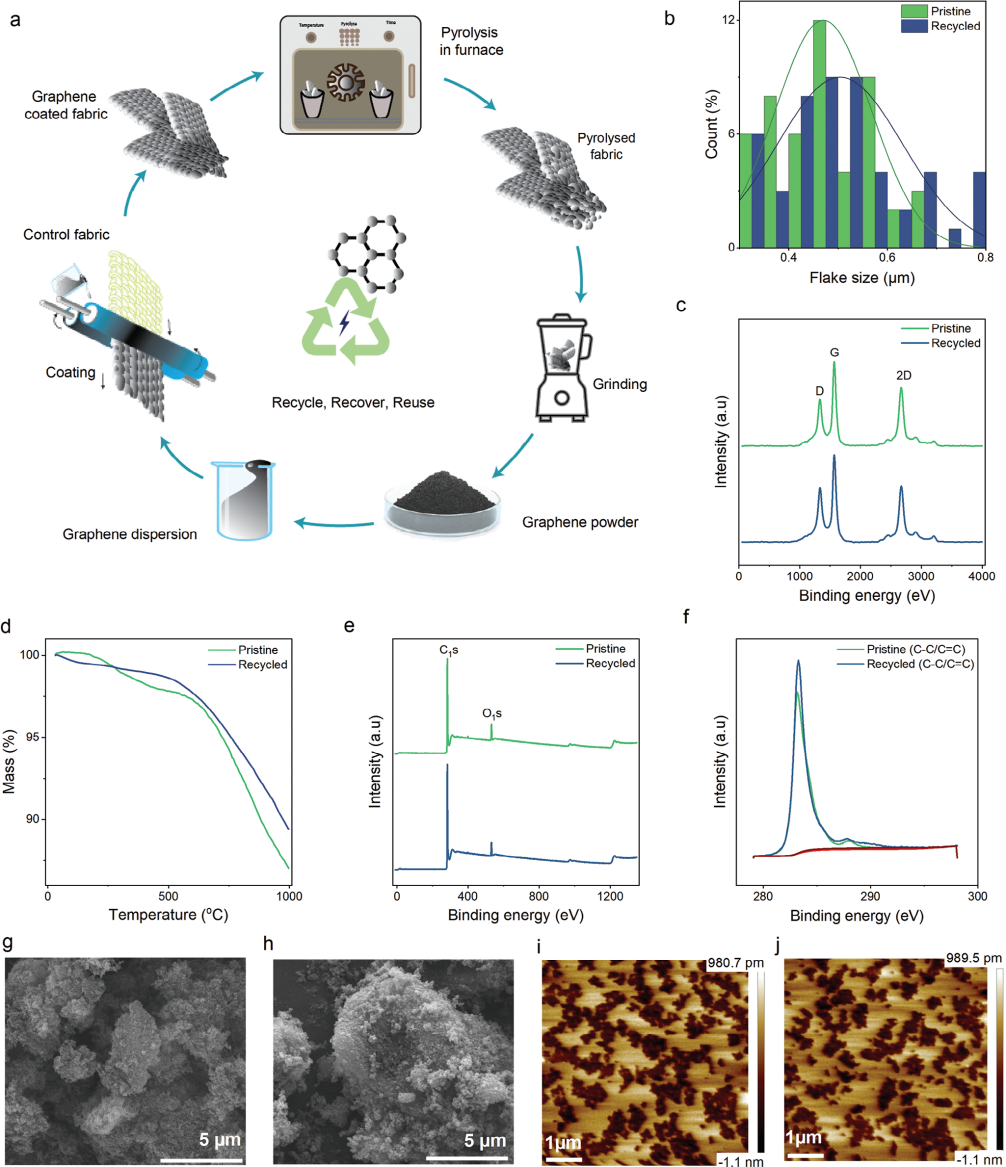
Closed‐loop recycling of wearable e‐textiles. a) Schematic of recycling e‐textiles toward sustainability via pyrolysis‐grinding and reuse of the recovered materials. Characterization of pristine and recycled graphene flakes: b) Size distribution. c) Raman spectrum of flakes. d) Plots of TGA‐ mass (%) with temperature change. e) Wide‐scan XPS spectra. f) High‐resolution XPS spectra. SEM image at X20000 magnification and 5 µm scale bar: g) pristine graphene, h) recycled graphene. AFM image of height sensor at 5 µm scale bar: i) pristine graphene, j) recycled graphene.

After that, we explored the recycling method to repurpose wearable e‐textiles by employing a two‐step process: pyrolysis followed by grinding, as depicted in **Figure** **2**a. Pyrolysis is the foundation of this approach, effectively upcycling discarded materials which transforms waste feedstocks of an inhomogeneous and complex nature into value‐added products (i.e., waste upcycling).^[^ ^38^, ^39^, ^40^ ^]^ Our procedure entailed exposing graphene‐coated Tencel fabric electrodes to intense heat in a furnace, maintaining an inert environment and absence of oxygen at a temperature of 1000 °C for 60 min to repurpose wearable e‐textiles. Tencel fibers require 300–600 °C to break down, according to earlier research,^[^ ^41^ ^]^ notably beyond 330 °C, with complete decomposition occurring between 385 and 460 °C.^[^ ^42^ ^]^ On the other hand, the ideal temperature range for graphene pyrolysis lies between 900 and 1100 °C,^[^ ^43^ ^]^ making 1000 °C an appropriate middle ground for processing both materials effectively. An inert atmosphere in the furnace was provided to prevent oxidation, control impurities, and maintain stable conditions of graphene‐coated textiles. Given the distinct pyrolysis temperatures required for lyocell and graphene, a trade‐off at 1000 °C for an hour was chosen under the maximum utilization of the facility range. This method not only allows for the recycling of materials, thus reducing waste but also improves the efficiency of resource use through the thermal breakdown of waste materials into simpler constituents in the absence of oxygen to produce reusable goods like biochar in line with circular economy principles leveraging the unique characteristics of graphene and cellulose‐based Tencel to produce carbon products of higher purity. The pyrolyzed fabric was then ground into a fine powder at the end of the process, after which the characteristics of these recaptured powder materials were thoroughly investigated and compared with the pristine graphene powder and then they were reused to fabricate wearable e‐textile devices. By this strategy, we ensure the sustainable use of resources by allowing materials to be recovered and reused continuously via a closed‐loop recycling process, reducing the need for virgin materials.

## 3 Recycled Materials from Wearable E‐Textiles

Figure 2b demonstrates that the average lateral size of recycled material flakes is ≈0.52 µm, and that of the biomethane‐derived (pristine) graphene flakes was found to be ≈0.47 µm.  Figure 2c shows a Raman spectrum of the pristine‐graphene flakes and recycled‐material flakes D peak at ≈1335 cm^−1^ and ≈1338 cm^−1^ indicating low defect density, G peak at ≈1570 cm^−1^ and ≈1570cm^−1^ and an asymmetric 2D band at ≈2672 cm^−1^ and ≈2669 cm^−1^ indicating a crystalline structure in both flakes, respectively, a typical spectrum for LPE graphene, with the characteristic D peak at ≈1350 cm^−1^, G peak at ≈1582 cm^−1^ and an asymmetric 2D band at ≈2730 cm^−1^.^[^ ^44^ ^]^ To investigate their dependency on thermal properties, the samples were subjected to thermal disintegration up to 1000 °C in the nitrogen atmosphere. The Thermogravimetric analysis (TGA) graph in Figure 2d shows that both pristine and recycled material remain thermally stable up to about 600 °C, however, considerable breakdown takes place at higher temperatures. The mass has not decreased to zero by the end of the temperature range, remarking that some residue remains. This residue could be any carbonaceous material that has not completely broken down even at 1000 °C. However, the chemical composition and phase state of the pristine graphene and recycled material flakes were examined using X−ray photoelectron spectroscopy (XPS) measurements. As seen from the wide−scan spectra of graphene materials in Figure 2e, pristine graphene and recycled flakes contain a small amount of oxygen. Figure 2f demonstrates the high−resolution C1s spectra, which affirm peaks for both pristine‐ and recycled‐flakes, dominated by C─C/C═C in aromatic rings at ≈284 eV. A peak at ≈288 eV was seen in the high‐resolution XPS spectra, and it may be related to carbon atoms in particular bonding environments, like C═O or O─C═O groups in materials as a result of surface oxidation that occurs on a small scale during handling, storage, or processing.^[^ ^45^ ^]^ Exposure to air can cause slow surface oxidation, which can result in the generation of these groups even under controlled conditions, despite their absence in the ideal structure.^[^ ^46^ ^]^ Because XPS is so sensitive to surface composition, it can frequently identify oxidation that is limited to the topmost layers.^[^ ^47^, ^48^ ^]^ Furthermore, inadequate purification or residual impurities from the synthesis process could be a factor in the existence of oxygenated species in pristine materials as supplied.^[^ ^49^, ^50^ ^]^ Surface oxidation during pyrolysis under an inert atmosphere is generally unlikely, as the goal of an inert atmosphere (usually using gases like argon or nitrogen) is to prevent reactions with oxygen. However, trace amounts of oxygen may still be present in the system, which could lead to minor oxidation, especially at high temperatures. While pristine materials may exhibit minimal oxidation, it is almost impossible to fully prevent surface oxidation in real‐world environments. The small intensity of these peaks suggests that oxidation is largely confined to the surface and does not significantly impact the bulk material properties. Therefore, the minor presence of these functional groups can be considered a normal artifact of handling and processing, without implying substantial degradation or contamination of the material itself.

**Figure 3:**
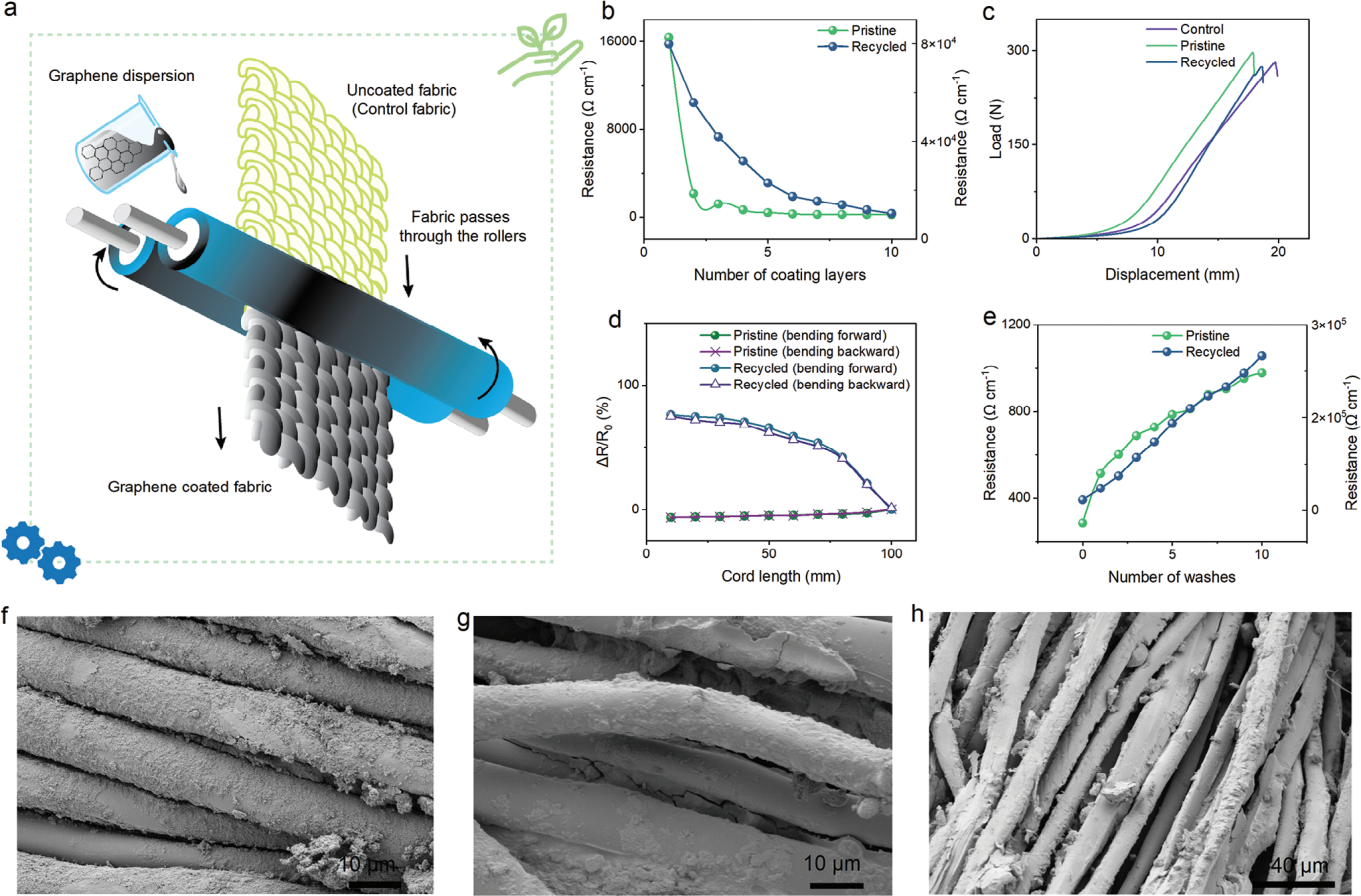
Scalable process for wearable e‐textiles with sustainable materials. a) Schematic of pad‐dry coating of a Tencel fabric with graphene dispersion. Comparison of the wearable e‐textiles properties between pristine‐ and recycled‐graphene‐based materials: b) Layer‐by‐layer variation in the electrical resistance of fabrics coated with graphene. c) Load–displacement curve of optimized graphene‐coated textiles in tensile testing compared to control fabric. d) The change in relative electrical resistance of graphene‐coated textiles during bending (forward and backward). e) The change in electrical resistance of graphene‐coated wearable e‐textiles with 10 number of washes. SEM images of f) pristine‐graphene‐coated fabric (X2500), g) recycled‐graphene‐coated fabric (X2500) and h) recycled‐graphene‐coated fabric (X1000).

**Figure 4:**
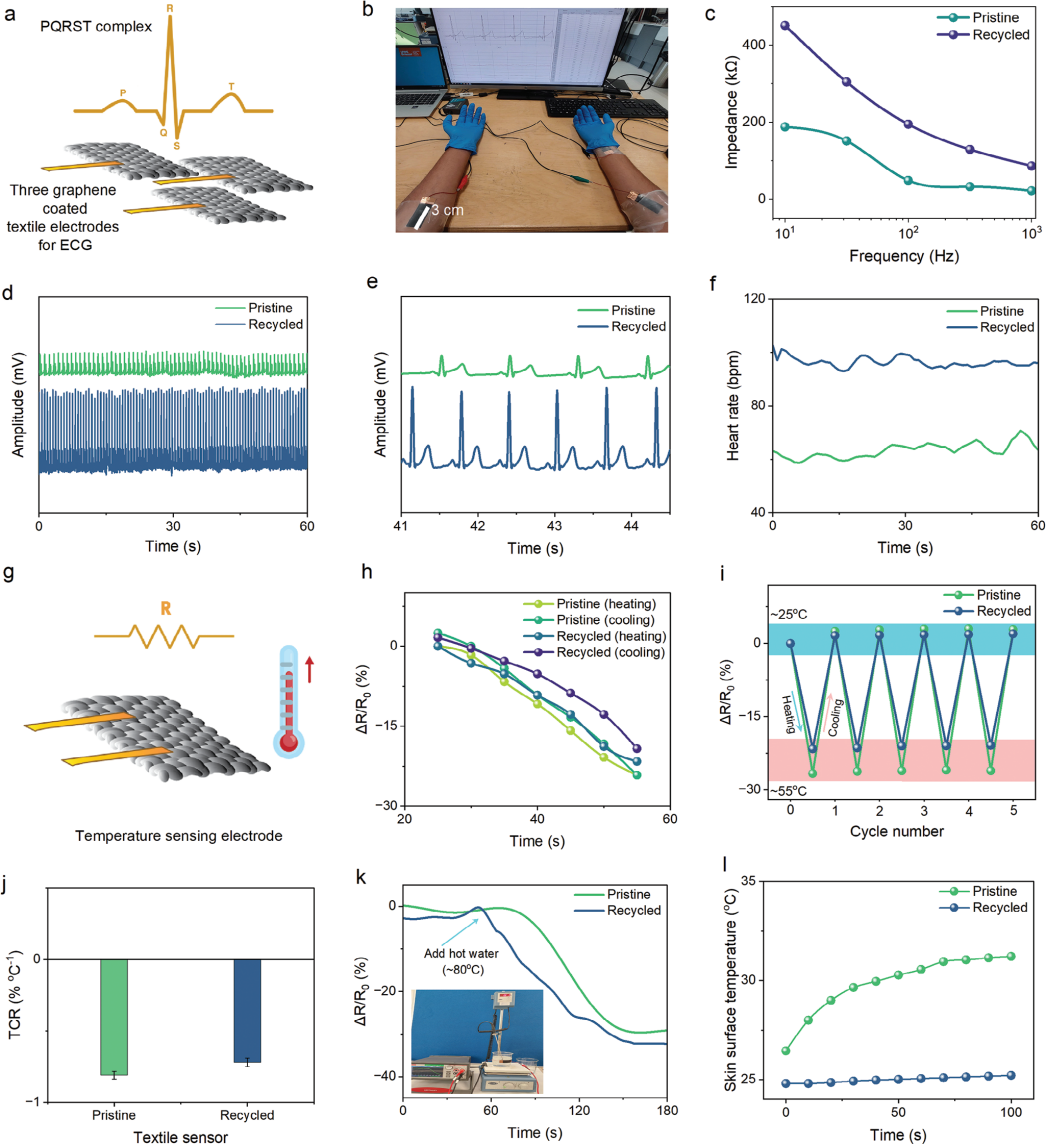
Wearable sensing applications for recycled e‐textiles. Comparison of pristine and recycled‐based devices: a) Schematic of graphene‐coated three textile electrodes for ECG signal detection. b) ECG electrodes positioning on the subject for capturing signal. c) Skin‐electrode contact impedance with frequency. d) ECG signal captured for the 60s of the subject (sitting) from graphene‐coated textile electrodes. e) Expanded version of (d) from 41 s to 44.4 s. f) Hear rate measured in bpm for 60 s of subject (sitting) from the QRS complex reading of (d) from graphene‐coated textile electrodes. g) Schematic of single graphene‐coated textile electrode for temperature sensing. h) Δ*R*/*R*_0_ change of graphene‐coated textile electrodes with heating and cooling on a hot plate. i) Δ*R*/*R*_0_ change of graphene‐coated textile electrodes for 5 cycles from low to high temperature via heating and from high to low temperature via cooling. j) TCR value of the textile‐based sensor, k) The effect of Δ*R*/*R*_0_ with time at adding hot water to a glass jar and the real‐time temperature sensing by the graphene‐coated electrode (inset), l) The response time to reach the maximum skin surface temperature.

**Figure 5:**
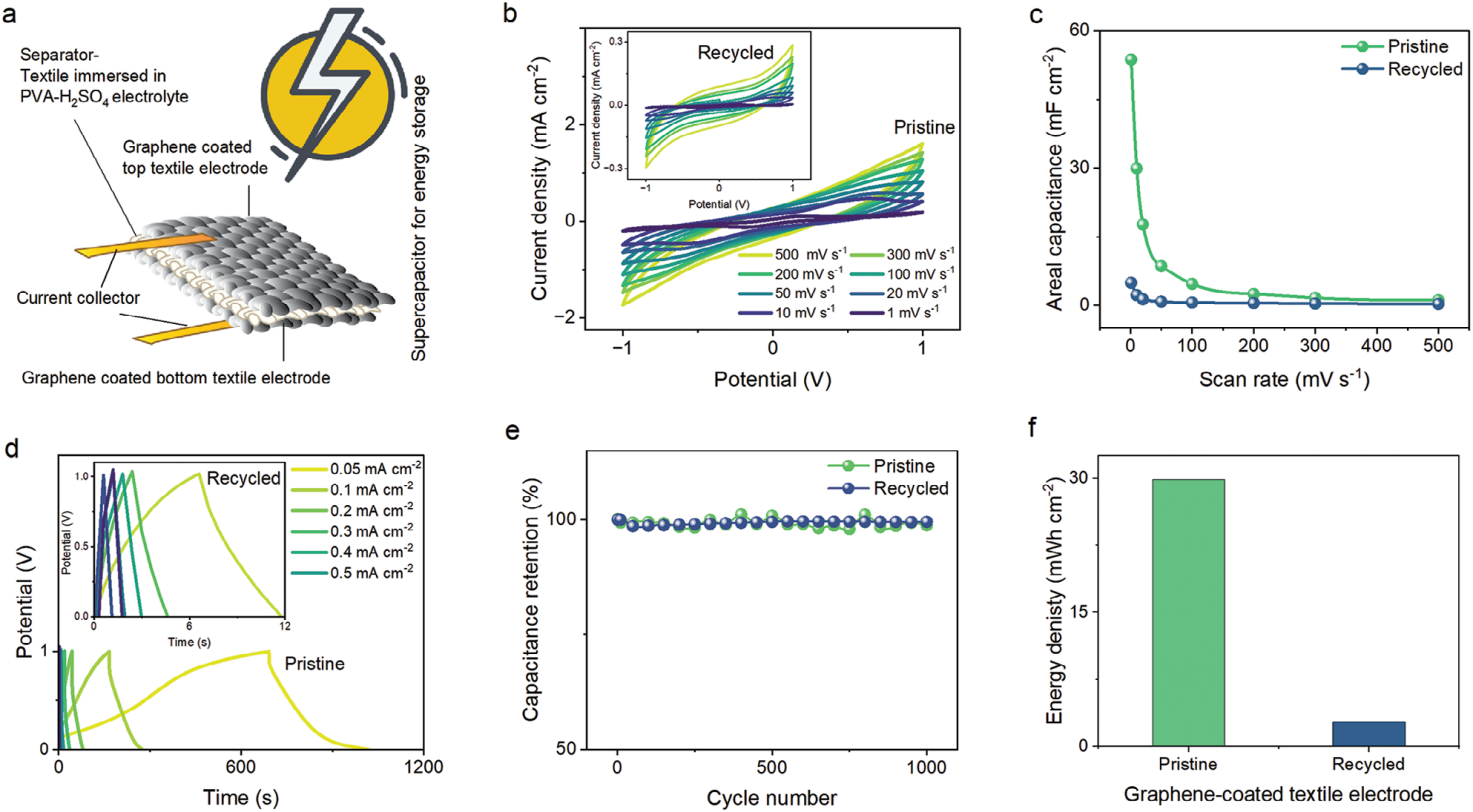
Wearable e‐textile devices of recycled materials in energy storage. Comparison of pristine and recycled‐based devices: a) Schematic of graphene‐coated textile electrodes used for energy storage as a supercapacitor. b) CV graph of graphene‐coated‐textile electrode‐based supercapacitor at different scan rates (outset‐pristine, inset‐recycled). c) Areal capacitance of graphene‐coated‐textile electrode‐based supercapacitor at different scan rates. d) GCD graph of graphene‐coated‐textile electrode‐based supercapacitor at different current densities (outset‐pristine, inset‐recycled). e) Capacitance retention (%) of pristine and recycled‐based supercapacitors after 1000 cycles. f) The changes in energy density of pristine and recycled‐based supercapacitors.

The structural morphology of the pristine‐ and recycled flakes in the high‐magnification SEM images was presented where the image has a great resolution, as evidenced by the X20000 magnification and 5 µm scale bar. The surface of the graphene flakes seems to be rough and textured, with aggregation of smaller particles, which is common in powdered graphene (Figure 2g). The recycled material exhibits a rough morphology typical of graphene materials, with a comparable textured surface and visible particles. However, the recycled flakes appear to be a little more clumped together, which could be because of the recycling process or variations in surface chemistry Figure 2h. In the Atomic Force Microscopy (AFM) images, pristine graphene and recycled material are examined at a 5 µm scale and show minor variations. The recycled sample seems darker, which might indicate that the thickness or surface structure has changed due to the recycling process. However, the recycled material dominantly displays characteristics comparable to the pristine graphene employed during the coating process in the beginning; thus it may be concluded that the material obtained through the pyrolysis process of graphene‐coated Tencel fabric is graphene‐like material denoted as recycled graphene in Figures 2, 3, 4, 5.

## 4 Reuse of Recycled Materials for Wearable E‐Textiles

In advancing sustainable wearable electronics, a pivotal achievement is the ability to construct devices entirely from recycled components, showcasing the potential of reusing materials for wearable e‐textiles. Our work derived conductive graphene‐like material through the recycling process, demonstrating Tencel‐graphene‐derived conductive recycled‐graphene powder as an example of the reuse of e‐textile waste for further fabrication. This method exemplifies a circular approach to managing the lifecycle of materials, particularly through the conversion of thermal breakdown products into coating dispersion. The dispersion was used for coating the Tencel fabric to fabricate textile electrodes (**Figure** **3**a), thereby minimizing waste and promoting the 4R principle (reduce, reuse, recycle, recover) in wearable technology.

The findings presented in Figure 3b reveal an apparent correlation between the number of coating layers and the structure's electrical resistance, where the electrical resistance of both pristine‐ and recycled‐graphene‐coated fabrics decreases with the number of coating layers, respectively. However, the pristine‐graphene‐coated fabric shows a more significant decrease in resistance, indicating better conductivity. After applying a single layer of pristine‐graphene dispersion coating, the fabric's resistance was found to be ≈16 400 Ω cm^−1^ by measuring with a digital multimeter. Remarkably, following the second coating layer, the resistance significantly decreased by ≈87%, reaching ≈2140 Ω cm^−1^. The resistance reduces with an increase in graphene layer count, reaching an average resistance of ≈286 Ω cm^−1^ for up to six graphene layers, whereas recycled graphene‐coated fabric possesses ≈17 500 Ω cm^−1^. In this study, resistance levels of pristine‐graphene‐coated textiles stabilized after the sixth layer, indicating a saturation point in the coating process, whereas in recycle‐graphene‐coated fabric, after six layers, its resistance slightly decreased but was not that significant. The initial layers were absorbed by the fibers, reducing resistance, but subsequent layers formed a conductive film on the fiber surface, as confirmed by SEM analysis. The pressure during the coating process enhanced the connectivity of graphene flakes, thus lowering resistance. Optimizing the process, the sixth layer provided the best balance of conductivity like other studies^[^ ^51^ ^]^ and for further tests, this optimized layer was used to make it more comparable within the same number of coating layers for pristine‐recycled. SEM images illustrate the deposition of material into the fabric for both pristine and recycled‐based coated fabric in Figure 3f–h. Particle separation could increase resistance; thus, it was important to carefully control the curing temperature to maintain conductivity. It was found that 100 °C for 7 min provided the best settings in this study as showing the least electrical resistance (≈280 Ω cm^−1^) over other times and temperatures in Figure S1a, Supporting Information.

Although recycled graphene retains its conductive qualities, the conductivity is much lower than that of pristine graphene layers, as evidenced by the noticeable rise in resistance. This might be the result of several factors, including contaminants, or structural changes to the graphene brought about by the recycling procedure. However, recycled graphene could be a feasible material for wearable e‐textile applications where ultra‐high conductivity is not required because it still shows conductivity. Figure 3c illustrates the tensile test, showing the force required to displace the fabric. The graph compares the mechanical behavior of three fabrics: control (uncoated fabric), pristine (supplied graphene‐based‐coated fabric), and recycled (recycled‐material‐based‐coated fabric). The pristine fabric demonstrates the highest load‐bearing capacity, indicating superior mechanical strength due to the graphene coating. It reaches a peak load of around 300 N at approximately 20 mm of displacement, outperforming both the control and recycled samples. The recycled fabric shows improved performance over the control, with a higher load‐bearing capacity, though it does not reach the same strength as the pristine fabric. This indicates that while the recycling process retains much of the coating's beneficial properties, the pristine graphene‐based coating offers the most significant enhancement. Overall, the results highlight the effectiveness of both commercial and recycled coatings in improving fabric strength compared to the uncoated control fabric. Both the pristine‐ and recycled‐graphene‐coated fabrics demonstrate a similar profile, indicating that the recycled graphene‐coated fabric retains approximately ≈92.54% of the mechanical properties compared to the pristine graphene‐coated fabric.

In Figure 3d (bending) and Figure S1b, Supporting Information (compression), the change in resistance (Δ*R*/*R*_0_) upon bending and compression shows that both pristine‐ and recycled‐graphene‐coated fabrics maintain consistent electrical properties under physical deformation. There is a moderate increase in resistance change for the recycled graphene as the overall electrical resistance was higher. Last, Figure 3e shows the change in resistance with the number of washes. The resistance of the recycled graphene‐coated fabric increased by almost ≈2 times after the first wash, compared to an approximate ≈1.80‐fold rise for the pristine graphene‐coated fabric. The resistance of the pristine‐graphene‐coated fabric grew by roughly ≈3.42 times after ten washes, while the recycled‐graphene‐coated fabric increased by roughly ≈14.29 times. When compared to pristine graphene‐coated fabric, the resistance of recycled graphene‐coated fabric increases with each wash cycle. This shows that washes will cause modifications to the recycled graphene, which could eventually compromise the durability and functionality of wearable e‐textiles made of recycled graphene. Overall, there is an apparent distinction in the electrical performance after recycling, especially after washing, even though the recycled graphene still has many mechanical and electrical properties of the pristine graphene. This difference may be caused by modifications in the graphene structure or its interaction with the fabric after pyrolysis.

## 5 Wearable Applications of Recycled E‐Textiles

### 5.1 Wearable Sensing Applications

The efficient use of recovered materials was demonstrated, aligning with circular economy principles^[^ ^52^, ^53^ ^]^ by keeping resources in use in multifunctional devices and encouraging upcycling. ECG sensing of the heart was performed using textile electrodes of eco‐friendly materials: pristine and recycled‐graphene‐coated Tencel fabric (**Figure** **4**a), respectively, to demonstrate functional sensing. These electrodes, positioned on either side of the wrist and upper forearm, measured the electrical activity caused by the heart's pumping action where each electrode area was 3 cm × 2 cm (Figure 4b). This was used to determine heart rate, which is important for various clinical and fitness applications of wearable technologies.^[^ ^54^ ^]^ Figure 4d provides an example of a collected signal demonstrating the possibility of obtaining high‐quality ECG recordings. This study revealed that electrodes made from pristine materials recorded a heart rate of 64 bpm (Figure 4f) with a regular R–R interval of ≈0.89 s (as shown in the extended version of Figure 4d in Figure 4e), producing well‐defined PQRST complexes in ECG signals.^[^ ^55^ ^]^ In contrast, electrodes fabricated from recycled materials exhibited a heart rate of ≈95 bpm with longer R–R intervals of ≈1.25 s, and the amplitude of the ECG signals was four times greater than that of the pristine. This aligns with the higher electrical resistance observed in the recycled electrodes.

The significant variation in heart rate and ECG signals between the pristine and recycled material‐based electrodes may be caused by differences in material composition, contact quality, and environmental sensitivities. These factors collectively impact signal acquisition, leading to the observed differences even when tested on the same human participant. Pristine electrodes have better conductivity and lower skin contact impedance compared to recycled electrodes. For instance, graphene‐coated textile electrodes showed an impedance range from 187.7 kΩ (at 10 Hz) to 21.55 kΩ (at 1 kHz), whereas recycled‐coated textile electrodes exhibited an impedance range from 450.8 kΩ (at 10 Hz) to 86.2 kΩ (at 1 kHz), Figure 4c. Higher impedance in recycled electrodes could lead to a higher amplitude of the recorded signal due to increased resistance. Additionally, recycled materials on fibers show slight surface unevenness having fiber material (Figure 3h) that affects signal quality, potentially increasing noise and altering the amplitude and R–R interval measurements.^[^ ^56^ ^]^ Despite these differences, both types of electrodes successfully captured the characteristic waveforms of ECG signals, indicating that sustainable material‐based electrodes are potential examples of reuse in recording cardiac activity, although recycled materials may alter the signal characteristics.

Pristine and recycled graphene‐coated textile electrodes (Figure 4g) in e‐textiles have comparable temperature‐responsive properties,^[^ ^57^, ^58^, ^59^ ^]^ with both showing a decrease in resistance as temperatures rose, indicated by negative TCR values (−0.81% °C⁻¹ for pristine and −0.72% °C⁻¹ for recycled) from the effect of Δ*R*/*R*_0_ within the temperature range 25–55 °C in Figure 4h, demonstrating that recovered materials for temperature sensing applications are not only feasible but also retain substantial functionality. This is because graphene integrates with substrates more seamlessly than metallic nanomaterials due to its electromechanical features^[^ ^60^ ^]^ and graphene's resistance varies with temperature, influenced by thermally activated charge carriers, potentially decreasing with rising temperature as charge carrier mobility increases. However, the lower TCR observed in Figure 4j in the recycled graphene‐based sensor (−0.72% °C^−1^) than in the pristine graphene sensor (−0.81% °C^−1^) can be attributed to the differences in material quality and structure. The pristine graphene‐coated textile sensor, derived from a company source using biomethane waste, exhibits high electrical conductivity and a highly crystalline structure due to the controlled synthesis from a single, uniform source which may cause fewer defects and improved electron mobility,^[^ ^61^ ^]^ leading to a higher TCR. In contrast, the recycled sensor is made from pyrolyzed graphene‐coated fabric, which is a combination of two separate material sources: textile and graphene. This process introduces structural defects, such as residual fibers in the nanoparticles and irregular graphene layers, which may impair electron transport and reduce the TCR. Studies have shown that the presence of impurities and defects in graphene,^[^ ^62^ ^]^ especially when derived from mixed or recycled sources, can significantly decrease its electrical and thermal performance, thereby explaining the lower TCR in the recycled sensor. Moreover, both types showed consistent performance over multiple heating and cooling cycles, with minimal variation in resistance changes, indicating reliable repeatability as shown in Figure 4i. For real‐time observation of how the resistance changed when hot water (≈80 °C) was added, a textile electrode was firmly attached to a glass jar (Figure 4k, inset). This showed rapid reaction speeds as seen in Figure 4k. The resistance varies, with recycled showing a falling trend with a very high slope and pristine showing an evenly decreasing trend. For both pristine and recycled, steady resistance is reached after ≈150 and ≈120 s, respectively. Also, within the response time of 100 s to reach the maximum skin surface temperature, the pristine and recycled electrodes were tested with a reference kit, where the pristine was able to reach ≈31 °C and recycled at ≈25 °C (Figure 4l). At different stimuli in real time, recycled electrodes maintained effective performance, highlighting the viability of upcycling in preserving functionality.

### 5.2 Wearable Supercapacitor Applications

To demonstrate sustainable wearable e‐textiles by pristine and recycled, a sandwich‐shaped symmetric textile supercapacitor was developed (**Figure** **5**a) using two optimized identical graphene‐coated electrodes and a PVA‐H_2_SO_4_ gel electrolyte, evidenced by extensive cyclic voltammetry (CV) and galvanostatic charge‐discharge (GCD) analyses. Constructed with identical graphene electrodes and a PVA‐H_2_SO_4_ gel electrolyte, the device showcased significant double‐layer capacitance, evident from the broad CV curves across a wide voltage (−1.0–1.0 V) range (Figure 5b), highlighting graphene's potential in energy storage applications for both pristine and recycled. The comparison between supercapacitors made from pristine and recycled graphene‐coated electrodes reveals a significant difference in performance, with the pristine achieving a capacitance of 53.73 mF cm⁻^2^, vastly outperforming the recycled's 4.92 mF cm⁻^2^ at a 1 mV s⁻¹ scan rate (Figure 5c). The recycled graphene's shorter discharging time (6 s) compared to the pristine's (324 s) in Figure 5d indicates faster energy release but lower overall energy storage, giving an energy density of 2.73 mWh cm^−2^ whereas pristine shows 29.85 mWh cm^−2^ in Figure 5f. Despite this, the recycled graphene maintains commendable durability, retaining ≈94% capacitance after 1000 cycles, slightly below the pristine's ≈98% (Figure 5e). These findings highlight the potential of upcycling in wearable e‐textiles for supercapacitors, offering a sustainable approach to material reuse with reasonable performance retention, crucial for eco‐friendly and cost‐effective energy storage solutions^[^ ^63^, ^64^, ^65^, ^66^, ^67^ ^]^ in wearable technologies.

## 6 Conclusion

The study highlights the efficacy of upcycling in wearable e‐textiles, using both pristine‐ and recycled‐graphene‐coated fabrics for ECG sensing and energy storage, showcasing functional sensing and comparable temperature‐responsive properties. While pristine electrodes offer higher performance, recycled materials still retain significant functionality, demonstrating the feasibility of sustainable practices in wearable technology. The recycled graphene's commendable durability in supercapacitors, despite lower capacitance compared to pristine, emphasizes upcycling's role in creating eco‐friendly, cost‐effective solutions. This approach aligns with sustainability goals, offering practical EoL solutions for wearable e‐textiles by maintaining functionality through upcycling, contributing to the advancement of wearable technology by repurposing waste into useful, even if the recycled doesn't surpass the original in terms of performance. However, the emphasis is on reducing waste and extending the lifecycle of materials, which still aligns with the principles of sustainability and circular economy, even if the performance aspect is slightly compromised.

To conclude, this study demonstrates a significant advancement in sustainable wearable technologies offering a recycling strategy. The flexible scalable e‐textiles represent versatility using material recovery and repurposing by recycling, promoting circularity. Sustainable techniques for reusing textile electrodes comprising substrates and conductors have been devised, doing away with the requirement for complex processes or purification. The developed e‐textiles show multifunctionality in wearable technology, including electrodes for ECG, and temperature sensing prominently. This work paves the road for a more environmentally friendly future in electronic wearables by advancing a circular economy and adhering to green methods in addition to addressing environmental impact.

## 7 Experimental Section

### Materials

The graphene powder (0.2 ± 0.05 µm lateral size, 130 ± 5 m^2^ g^−1^ specific surface area, combustion elemental analysis C > 97.5%, O < 2.5%) was supplied by Levidian (UK). Poly(vinyl alcohol) (PVA) (molecular weight: 31000–50000, 98–99% hydrolyzed) and sulfuric acid (H_2_SO_4_) (puriss, 95–97%) were purchased from Merck, UK. Sodium deoxycholate (≥97% (titration), SDC powder was purchased from Merck, UK and used as received. 100% Lenzing Tencel fabric was donated from Nice Denim Mill Ltd., Bangladesh.

### Material Characterization

To obtain Raman spectra, AFM, SEM, and optical microscopic images of graphene powder and recycled powder, clean samples were prepared on oxidized Si substrates (with 290 nm thick SiO_2_ layer) by drop casting. FEI Quanta 650 Field Emission Scanning Electron Microscope (SEM) (Hitachi Regulus 8100) were used to assess the lateral particle size. Raman spectra were captured using LabRam HR Evolution (Weave length: 633; Test range: 50–4000). A Thermo ESCALAB 250XI was used for XPS analysis for the elemental composition and their chemical state. The concentration of the print ink was measured by a Camspec M509T spectrophotometer. A Rigaku (Temperature: room: 1000 °C; Gas used: Nitrogen; Temperature ramp: 20 °C min^−1^) was used for the thermogravimetric analysis (TGA) of recycled materials. The morphology of the recycled material was studied with atomic force microscopy (AFM) (Bruker dimensions Icon, Germany).

### Coating Dispersion Preparation

Graphene dispersion was used for the pad‐dry‐cure of textiles for the fabrication of e‐textile electrodes. SDC was used as a surfactant to effectively stabilize the dispersion for further coating. The dispersion was prepared by mixing 10 g of graphene powder and 1 g of SDC into 100 mL of DI water, ensuring a uniform mixture through magnetic stirring for 30 min.

### Pyrolysis and Grinding

A 10 mg sample of graphene‐coated fabric was stored in each ceramic crucible and then placed inside a GPCMA (modified atmosphere furnace) retort furnace for heat treatment (Carbolite GERO Ltd.). The argon flow rate was 15 L min^−1^ for a 15 min purge at the beginning to vent out the impurities, and the temperature was then raised to 1000 °C. The sample was then pyrolyzed at 1000 °C in an inert environment (argon gas) for 60 min. The furnace was allowed to cool for 16 to 20 hours after the predetermined residence time, and then the pyrolyzed sample was gently removed. After pyrolysis, recycled materials were ground using a 200 W mini grinder.

### Pad‐Dry Coating of Textiles

Textile fabrics were padded (one dip and one nip) through graphene dispersions to achieve a wet pick‐up of ≈80% of the weight of the fabric. The weight pick‐up is measured by following the equation.

(1) $$\begin{array}{ccc} & & \mathrm{Pick}-\mathrm{up}\;\;\left(\%\right)\\ & & =\frac{\mathrm{The}\;\mathrm{weight}\;\mathrm{of}\;\mathrm{wet}\;\mathrm{padded}\;\mathrm{fabric}-\mathrm{the}\;\mathrm{dry}\;\mathrm{weight}\;\mathrm{of}\;\mathrm{control}\;\mathrm{fabric}}{\mathrm{The}\;\mathrm{dry}\;\mathrm{weight}\;\mathrm{of}\;\mathrm{control}\;\mathrm{fabric}}\\ & & \times 100 \end{array}$$

A vertical and horizontal (350) 2 Bowl BVHP PADDER (Roaches, UK) with a variable number of layers was used for the coating process. One padding pass and one drying pass are completed in each cycle, carried out at a speed of 1 m min^−1^ at a pressure of 0.74 bar. After the coating, the fabric was then dried at 80 °C for 5 min in a Mini Thermo Oven Type 350 Special (Roaches, UK) to fix the graphene firmly onto the fabric before further evaluation and characterization. The number of padding cycles indicated the number of coating layers applied. It is noteworthy that, textile fabrics were padded with multiple (1 to 10) padding passes to optimize their electrical conductivity. To characterize the surface functionalities and elemental composition and identify optimum curing conditions^[^ ^68^ ^]^ for sustainable fabrics at different times and temperatures were assessed also.

### Fabrication of Supercapacitor

The supercapacitor was made in a sandwich configuration, wherein the coated samples (1 cm × 1 cm) served as electrode materials for both the top and bottom layers. Between these electrode layers was the intervening separator (Figure 5a), which was made of Tencel fabric submerged in a hydrogel‐polymer electrolyte PVA doped with H_2_SO_4_. Connecting wires were connected to the fabric surface in order to collect current using silver electronic. The ends of each electrode, however, had wires affixed to them to guarantee proper electrical contact with the measurement workstation. The PVA‐H_2_SO_4_ gel electrolyte was prepared as follows: 1 g of H_2_SO_4_ was added to 10 mL of DI water after 1 g of PVA. After that, the mixture was heated to about ≈85 °C and agitated until the clear solution was achieved. The electrolyte was kept in its sandwiched state by drop‐casting it onto the separator fabric. After that, it was allowed to dry naturally for the entire night in order to make sure that the electrolyte completely wet the electrode and to allow any extra water to evaporate.

### Tensile Strength Testing

Graphene contributes to the development of sustainable products that have the potential to reduce the negative environmental impact of conventional materials while also offering improved mechanical properties.^[^ ^69^ ^]^ Using a Testometric material tensile machine (UK), load cell 100, the tensile strength of the uncoated and graphene‐coated test specimens (pristine and recycled) was obtained at a speed of 100 mm min^−1^. Before the start, the specimen size was prepared by fraying down the fabric from both sides to give a central width of 2 cm and maintaining a distance between jaws of 10 cm as gauge length.

### Flexibility Testing

By following previously reported methods^[^ ^70^, ^71^ ^]^ the flexibility of coated e‐textiles was evaluated. Various cord lengths were used to measure the change of resistance of coated (10 cm × 1 cm strip) during bending (concave down) and (compression (concave upward). A Win‐Test tensile tester (Testometric, UK) was used to control the cord length both in the forward and reverse directions for both bending and compression tests. The change in the electrical resistance of the textiles during bending and compressions was captured using a Keithley digital multimeter (DMM7510).

### Washability Testing

The washability assessment of coated fabric in various washing cycles (1–10) following BS EN ISO 105 C01 method,^[^ ^72^ ^]^ at 40 °C for 30 min, European Colourfastness Establishment (ECE) Reference Detergent was used with magnetic stirring to reproduce the action of hand washing, as it is recommended to handle 100% Tencel fabric at low temperature.

### ECG Measurement

In medical practice, a standard ECG involves the placement of 12 leads on the body, with six leads positioned on the chest to directly monitor heart activity. This research focuses on developing coated electrode patches for use in the laboratory setting to create a simplified version of the ECG that is easily interpretable by the general population. Given the experimental nature of the work showing the sensing capability of electrodes, a 3‐lead setup from an ECG sensor device was used, which allows for effective signal capture. The wearable textile electrodes were fabricated by coating optimized layers, and at the edges of the textile, one connecting wire to each electrode patch was attached for ECG signal and heart rate measurement with the Go Direct EKG device (USA). This device measures electrical activity in the heart and electrical signals produced during muscle contractions. The default active channel was built‐in optimized for recording ECGs, utilizing a low‐pass filter with a 22.5 Hz ‐3 dB cutoff and ‐80 dB attenuation above 50 Hz. For this device, the 3‐lead ECG tracing settings were used, and prepared electrode patches were attached to the subject, as shown in Figure 4b, as per the guidelines.^[^ ^73^ ^]^ A single patch was placed on the inside of the right wrist, on the inside of the right upper forearm (distal to the elbow), and the inside of the left upper forearm (distal to the elbow), and the reference device's clips were connected to the electrode tabs. The subject was in a relaxed position (sitting) in a chair for ECG and heart rate measurements, and the ECG channel used a low‐pass filter optimized for recording ECGs. This is the default channel that is active when the sensor is connected. The heart rate channel detected QRS waveforms and used that data to calculate the heart rate in beats per minute (bpm). The sampling window for this calculation was 6s, and the value was updated every second.

### Temperature Sensor

A resistance measurement setup was used together with a hotplate to observe the heating and cooling cycle and assess the resistance dependence on temperature for Figure 4b,c. A digital multimeter (Keithley) was used in the resistance measurement setup, and the sensor was placed on a hotplate to drive the heating/cooling process. The two connecting wires to the temperature sensor electrode in the sensitivity measurement setup connection were embedded in the fabric to prevent layer scratching. The sensitivity of the temperature sensor was defined by its temperature coefficient of resistance (TCR) and can be calculated from Equation 2.

(2) $$\mathrm{TCR}\;\left({\%}^{\circ }C^{-1}\right)\;=\frac{\left(R-R_{0}\right)}{R_{0}\times \Delta T}\;\times 100$$

*R* and *R*_0_ are the resistance at the sensor's measured temperature and room temperature, and ∆*T* is the change in applied temperature. Furthermore, the electrode was attached to the outside of a glass jar to confirm the hot water temperature over it. The subject also wore the coated textile electrode to measure the sensitivity in skin surface temperature. The coated electrode's response time upon skin surface contact was measured with the connection with commercially available circuit module MAX30205.

### Supercapacitor Characterization

The fabricated textile‐based supercapacitor's electrochemical properties were investigated using galvanostatic charge/discharge (GCD) and cyclic voltammetry (CV) testing. The Iviumstat electrochemical interface was used to carry out these electrochemical analyses. The potential range of −1.0 to 1.0 V was used for the CV and GCD investigations, which included a range of scan rates and current densities. This process was intended to calculate critical metrics that are necessary to describe an energy storage device's performance using the following formulas.

Charge storage ability per unit area: Areal capacitance [F cm^−2^],

(3) $$C_{A}=\frac{A}{2\mathrm{saV}}\;$$

Amount of energy able to deliver: Energy density [Wh kg^−1^],

(4) $$E\;=\frac{1}{2}\;{\mathrm{CV}}^{2}$$

How faster is the energy to deliver: Power density [W kg^−1^],

(5) $$P\;=\frac{E}{t}\;$$

where, *C* = capacitance, *V* = voltage window, *t* = discharge time, *A* = integrated area of the CV curve, *s* = scan rate (mV s^−1^), *m* = mass of the electroactive material on both electrodes, *a* = area of the electrode.

## Conflict of Interest

The authors declare no conflict of interest.

## Supporting information



Supporting Information

## Data Availability

The data that support the findings of this study are available from the corresponding author upon reasonable request.
