# Supplementary material for: Closed‐Loop Recycling of Wearable Electronic Textiles
Source: Small. 2024 Oct 2;20(50):2407207. doi: 10.1002/smll.202407207 (PMC11636061; doi:10.1002/smll.202407207)
Supplement: Supplementary file 1 — Supporting Information [file SMLL-20-2407207-s001.docx]

**Closed-loop recycling of wearable electronic textiles**

Marzia Dulal, Shaila Afroj^*^, Md Rashedul Islam, Minglonghai Zhang, Yadie Yang,

Hong Hu, Kostya S. Novoselov and Nazmul Karim^*^

M. Dulal, Dr. M. R. Islam, Dr. S. Afroj and Prof. N. Karim,

Centre for Print Research, The University of the West of England Bristol, BS16 1QY, UK.

M. Dulal

Department of Textile Engineering Management, Bangladesh University of Textiles (BUTEX), Tejgaon Industrial Area, Dhaka-1208, Bangladesh.

Dr. S. Afroj

Faculty of Environment, Science and Economy, Department of Engineering, University of Exeter, EX4 4QF Exeter, United Kingdom UK. E-mail: s.afroj@exeter.ac.uk.

Dr. M. R. Islam

Department of Wet Process Engineering, Bangladesh University of Textiles (BUTEX), Tejgaon Industrial Area, Dhaka-1208, Bangladesh.

Dr. M. Zhang, Dr. Y. Yang and Prof. H. Hu

School of Fashion and Textiles, the Hong Kong Polytechnic University, Hong Kong.

Prof K. S. Novoselov

Institute for Functional Intelligent Materials, Department of Materials Science and Engineering, National University of Singapore, Singapore 117575, Singapore

Prof. N. Karim

Nottingham School of Art and Design, Nottingham Trent University, Shakespeare Street, Nottingham, NG1 4GG, UK. Email: nazmul.karim@ntu.ac.uk

Prof. N. Karim

Department of Fashion and Textiles, University of Southampton, Southampton SO23 8DL, UK.

**Wearable e-textiles and their device performance**

**
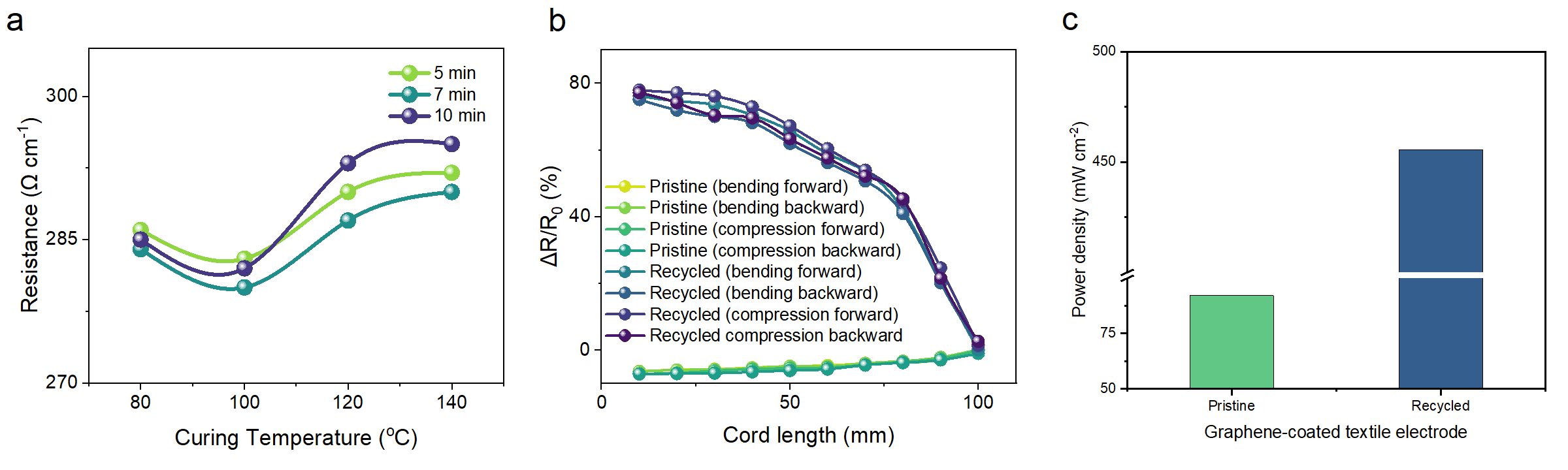
**

Figure S1 Wearable textiles and their device performance. a) The change in the electrical resistance of optimised layer-graphene-coated textiles for curing time-temperature optimization. b) Variation in resistance of pristine and recycled graphene-coated textiles during bending and compression. c) The changes in power density of pristine and recycled-based supercapacitors.

**Skin-contact impedance**

The prepared electrodes' tight-fitting pressure is applied on the skin by the flexible film, where the applied pressure helps maintain a stable electrode-skin contact and also lowers the electrode-skin contact impedance by reducing the air gap between the textile electrodes and the skin. Fitting results of the impedance spectra obtained on the skin surface of the arm with the electrodes. This study explores sustainable innovations in dry electrodes, contrasting them with traditional wet/gel electrodes known for excellent skin adhesion but causing discomfort during prolonged use. Developed dry electrodes on the wrist to measure skin-contact impedance crucial for ECG signal acquisition using a two-probe Iviumstat Electrochemical Interface were tested. After allowing for the moisture accumulation from sweat for 4-5 minutes, we recorded impedance in the 10 Hz to 1 kHz range. The findings reveal that while the gel electrodes exhibited impedance between 24.1 kΩ (at 10 Hz) and 3.1 kΩ (at 1 kHz), aligning with existing literature, graphene-coated textile electrodes showed a range from 187.7 kΩ (at 10 Hz) to 21.55 kΩ (at 1 kHz). Despite the higher impedance of our coated dry electrodes compared to reference gel types, they remain within the acceptable impedance spectrum for dry electrodes as large as several hundred kilo-ohms to mega-ohms,^1-5^validating their accuracy and dependability and viability for ECG monitoring with enhanced user comfort demonstrating the potential of our textile electrode *via* skin-electrode interface by meeting up requirements^5-7^ of wearer convenient flexible, effective ionic current flow and signal acquisition.


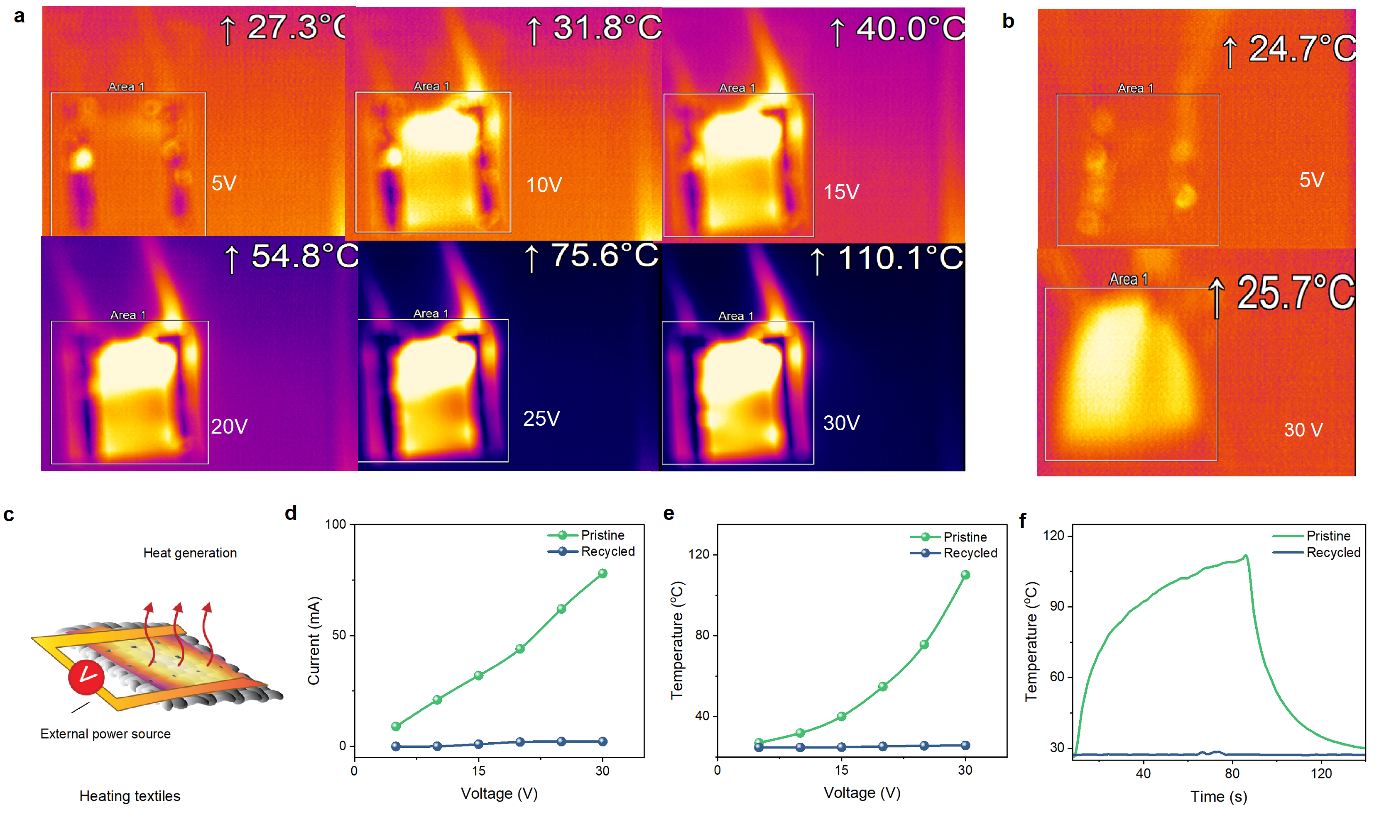


Figure S2 Heating textile elements. a) Thermal image of graphene-based heating textiles at different voltages: 5V, 10V, 15V, 20V,25V,30V for pristine sample. b) Thermal image of graphene-based heating textiles at different voltages: 5V, 30V for recycled sample. c) Schematic of graphene-coated heating textiles by applying external voltage. d) I-V curve and e) Graphene-coated heating textiles elements' surface temperature changes with the change of supplied voltage. f) Time-Temperature graph of graphene-coated heating textiles at 30V supplied voltages with the influence of power turns on and turn-off.

Table S1 Overview of performance comparison of some reported applications in the literature.

| **ECG sensor electrodes** | | | | | |
| --- | --- | --- | --- | --- | --- |
| Sl | Fabrication | Electrode position | Skin–electrode interface impedance | Mean RR interval length (s) | Ref |
| 1 | silver/silver chloride (Ag/AgCl) gelled electrodes |  | 118.7 kΩ for frequencies under 10 Hz | - | ^8^ |
| 2 | Carbon-based dry patch electrode | chest | 70.0 kΩ for frequencies under 10 Hz | - | ^8^ |
| 3 | graphene-clad textile electrode | - | 87.5 kΩ (at 10 Hz) to 11.6 kΩ (at 1 kHz). | - | ^9^ |
| 4 | conventional Ag/AgCl electrode | - | 50.9 kΩ (at 10 Hz) to 2.2 kΩ (at 1 kHz) | - | ^9^ |
| 5 | silver plated knitted fabric | ECG t-shirt | - | 0.898 ± 0.079 (sitting) | ^10^ |
| 6 | Polypyrrole-coated cotton | Forearms and ground on the ankle | 325 Sheet Resistance (Ω/sq) | - | ^11^ |
| 7 | Gel electrodes | wrist and left forearm and right forearm | 24.1 kΩ (at 10 Hz) and 3.1 kΩ (at 1 kHz) | ~0.87 (sitting) | Commercial |
| 8 | Graphene-coated Tencel fabric | wrist and left forearm and right forearm | 187.7 kΩ (at 10 Hz) to 21.55 kΩ (at 1 kHz) | ~0.89 (sitting) | This work |
| 9 | Recycled graphene-coated Tencel fabric | wrist and left forearm and right forearm | 450.8 kΩ (at 10 Hz) to 86.2 kΩ (at 1 kHz) | ~1.25 (sitting) | This work |
| **Temperature sensor** | | | | | |
| Sl | Materials and fabrication | Operating temperature range  (°C) | TCR  (% °C^-1^) | Response time -temperature with skin surface contact (s) | Ref |
| 1 | Layer-by-layer assembly of chitosan and carbon nanotube on cotton fabric | - | 0.471 | - | ^12^ |
| 2 | AgNWs/MXene/knitted fabric | - | 0.07 | - | ^13^ |
| 3 | Graphene-coated polypropylene | 30 - 45 | −0.17 | - | ^14^ |
| 4 | RGO/CNTs@PBT melting blown nonwoven fabric | 25 - 45 | −0.737 | - | ^15^ |
| 5 | Graphene-coated Tencel fabric | 25 - 55 | -0.81 | ~88 s to reach 31^o^C | This work |
| 6 | Recycled graphene-coated Tencel fabric | 25 - 55 | -0.72 | ~100 s to reach ~26^o^C | This work |
| **Heating textiles** | | | | | |
| Sl | Materials and fabrication | Temperature range (°C) | Applied voltage (V) | Recovery time (s) | Ref |
| 1 | cotton/CP - poly(3,4-ethylenedioxythiophene)-poly (styrene sulfonate) (PEDOT: PSS)/ dip-coating | ~46 | 25 | 1800 | ^16^ |
| 2 | MWCNTs Coated Cotton | ~ 90 | 10–60 | - | ^17^ |
| 3 | MWCNTs/Silk Fabric  micro-dissolution strategy | ~49.1 | 5–25 | - | ^18^ |
| 4 | PPy-Coated Lyocell Yarn | 30.5–106.6 | 1–6 | - | ^19^ |
| 5 | PPy/cotton, dipped | 25.5–168.3 | 1–16 | - | ^20^ |
| 6 | Graphene-coated Tencel fabric | 27-111 | 5-30 | 4-65 | This work |
| 7 | Recycled graphene-coated Tencel fabric | 24-26 | 5-30 | Not significant | This work |
| **Supercapacitor** | | | | | |
| Sl | Materials and fabrication | Areal capacitance | Energy density | Power density |  |
| 1 | dipping and drying of SWNTs ink on textiles  (1 M LiPF_6_ electrolyte) | 0.48F cm^-2^ | 20 Wh kg^−1^ | 10 kW kg^−1^ | ^21^ |
| 2 | printed on textiles using graphene oxide ink  and a screen-printing technique  (PVA-H_2_SO_4_ electrolyte) | 2.5 mF  cm^-2^ | - | - | ^22^ |
| 3 | all-in-one single fiber  rGO-GO-rGO  (region-specific reduction of graphene oxide (GO) fiber by laser irradiation  (0.1 M NaClO_4_ electrolyte) | 2 mF cm^-2^ | 2–5.4×10-^4^ Wh cm^-2^ | 3.6–9×10^-2^ W cm^-2^ | ^23^ |
| 4 | carbon fiber/MnO_2_ (CF/MnO_2_) and carbon fiber/MoO_3_ (CF/MoO_3_) fabricated using a simple electrodeposition method (1 M HCl electrolyte) | 4.86 mF cm^-2^  at 10 mV s^-1^ | 2.70 μWh cm^-2^ | 0.53 mW cm^-2^ | ^24^ |
| 5 | Graphene-coated Tencel fabric | 53.73 mF cm^-2^  at 1 mV s^-1^ | 29.85 mWh cm^-2^ | 92.13 mW cm^-2^ | This work |
| 6 | Recycled graphene-coated Tencel fabric | 4.92 mF cm^-2^  at 1 mV s^-1^ | 2.73 mWh cm^-2^ | 455.56  mW cm^-2^ | This work |

**References**

(1) Song, H. Y.; Lee, J. H.; Kang, D.; Cho, H.; Cho, H. S.; Lee, J. W.; Lee, Y. J. Textile electrodes of jacquard woven fabrics for biosignal measurement. *The Journal of the Textile Institute* **2010**, *101* (8), 758-770.

(2) Chi, Y. M.; Jung, T. P.; Cauwenberghs, G. Dry-Contact and Noncontact Biopotential Electrodes: Methodological Review. *IEEE Reviews in Biomedical Engineering* **2010**, *3*, 106-119. DOI: 10.1109/RBME.2010.2084078.

(3) Saleh, S. M.; Jusob, S. M.; Harun, F. K. C.; Yuliati, L.; Wicaksono, D. H. Optimization of reduced GO-based cotton electrodes for wearable electrocardiography. *IEEE Sensors Journal* **2020**, *20* (14), 7774-7782.

(4) Fish, R. M.; Geddes, L. A. Conduction of electrical current to and through the human body: a review. *Eplasty* **2009**, *9*.

(5) Lim, K.; Seo, H.; Chung, W. G.; Song, H.; Oh, M.; Ryu, S. Y.; Kim, Y.; Park, J.-U. Material and structural considerations for high-performance electrodes for wearable skin devices. *Communications Materials* **2024**, *5* (1), 49. DOI: 10.1038/s43246-024-00490-8.

(6) Kim, J.; Lee, M.; Shim, H. J.; Ghaffari, R.; Cho, H. R.; Son, D.; Jung, Y. H.; Soh, M.; Choi, C.; Jung, S. Stretchable silicon nanoribbon electronics for skin prosthesis. *Nature communications* **2014**, *5* (1), 5747.

(7) Li, Z.; Guo, W.; Huang, Y.; Zhu, K.; Yi, H.; Wu, H. On-skin graphene electrodes for large area electrophysiological monitoring and human-machine interfaces. *Carbon* **2020**, *164*, 164-170.

(8) Lee, J.-W.; Yun, K.-S. ECG Monitoring Garment Using Conductive Carbon Paste for Reduced Motion Artifacts. *Polymers* **2017**, *9* (9), 439.

(9) Yapici, M. K.; Alkhidir, T.; Samad, Y. A.; Liao, K. Graphene-clad textile electrodes for electrocardiogram monitoring. *Sensors and Actuators B: Chemical* **2015**, *221*, 1469-1474. DOI: https://doi.org/10.1016/j.snb.2015.07.111.

(10) Boehm, A.; Yu, X.; Neu, W.; Leonhardt, S.; Teichmann, D. A Novel 12-Lead ECG T-Shirt with Active Electrodes. *Electronics* **2016**, *5* (4), 75.

(11) Zhou, Y.; Ding, X.; Zhang, J.; Duan, Y.; Hu, J.; Yang, X. Fabrication of conductive fabric as textile electrode for ECG monitoring. *Fibers and Polymers* **2014**, *15*, 2260-2264.

(12) Zhang, Y.-X.; Li, Y.-D.; Du, A.-K.; Wu, Y.; Zeng, J.-B. Layer-by-layer assembly of chitosan and carbon nanotube on cotton fabric for strain and temperature sensing. *Journal of Materials Science & Technology* **2024**, *173*, 114-120. DOI: https://doi.org/10.1016/j.jmst.2023.07.025.

(13) Zhao, W.; Zheng, Y.; Qian, J.; Zhaofa, Z.; Jin, Z.; Qiu, H.; Zhu, C.; Hong, X. AgNWs/MXene derived multifunctional knitted fabric capable of high electrothermal conversion efficiency, large strain and temperature sensing, and EMI shielding. *Journal of Alloys and Compounds* **2022**, *923*, 166471. DOI: https://doi.org/10.1016/j.jallcom.2022.166471.

(14) Rajan, G.; Morgan, J. J.; Murphy, C.; Torres Alonso, E.; Wade, J.; Ott, A. K.; Russo, S.; Alves, H.; Craciun, M. F.; Neves, A. I. S. Low Operating Voltage Carbon–Graphene Hybrid E-textile for Temperature Sensing. *ACS Applied Materials & Interfaces* **2020**, *12* (26), 29861-29867. DOI: 10.1021/acsami.0c08397.

(15) Wang, N.; Sun, H.; Yang, X.; Lin, W.; He, W.; Liu, H.; Bhat, G.; Yu, B. Flexible temperature sensor based on RGO/CNTs@PBT melting blown nonwoven fabric. *Sensors and Actuators A: Physical* **2022**, *339*, 113519. DOI: https://doi.org/10.1016/j.sna.2022.113519.

(16) Jalil, M. A.; Ahmed, A.; Hossain, M. M.; Adak, B.; Islam, M. T.; Moniruzzaman, M.; Parvez, M. S.; Shkir, M.; Mukhopadhyay, S. Synthesis of PEDOT:PSS Solution-Processed Electronic Textiles for Enhanced Joule Heating. *ACS Omega* **2022**, *7* (15), 12716-12723. DOI: 10.1021/acsomega.1c07148.

(17) Rahman, M. J.; Mieno, T. Conductive Cotton Textile from Safely Functionalized Carbon Nanotubes. *Journal of Nanomaterials* **2015**, *2015*, 978484. DOI: 10.1155/2015/978484.

(18) Zhou, J.; Zhao, Z.; Hu, R.; Yang, J.; Xiao, H.; Liu, Y.; Lu, M. Multi-walled carbon nanotubes functionalized silk fabrics for mechanical sensors and heating materials. *Materials & Design* **2020**, *191*, 108636. DOI: https://doi.org/10.1016/j.matdes.2020.108636.

(19) Lv, J.; Dai, Y.; Xu, H.; Zhong, Y.; Zhang, L.; Chen, Z.; Sui, X.; Feng, X.; Wang, B.; Mao, Z. Transforming commercial regenerated cellulose yarns into multifunctional wearable electronic textiles. *Journal of Materials Chemistry C* **2020**, *8* (4), 1309-1318, 10.1039/C9TC05673D. DOI: 10.1039/C9TC05673D.

(20) Xie, J.; Pan, W.; Guo, Z.; Jiao, S. S.; Ping Yang, L. In situ polymerization of polypyrrole on cotton fabrics as flexible electrothermal materials. *Journal of Engineered Fibers and Fabrics* **2019**, *14*, 1558925019827447. DOI: 10.1177/1558925019827447.

(21) Hu, L.; Pasta, M.; La Mantia, F.; Cui, L.; Jeong, S.; Deshazer, H. D.; Choi, J. W.; Han, S. M.; Cui, Y. Stretchable, porous, and conductive energy textiles. *Nano letters* **2010**, *10* (2), 708-714.

(22) Abdelkader, A. M.; Karim, N.; Vallés, C.; Afroj, S.; Novoselov, K. S.; Yeates, S. G. Ultraflexible and robust graphene supercapacitors printed on textiles for wearable electronics applications. *2D Materials* **2017**, *4* (3), 035016.

(23) Hu, Y.; Cheng, H.; Zhao, F.; Chen, N.; Jiang, L.; Feng, Z.; Qu, L. All-in-one graphene fiber supercapacitor. *Nanoscale* **2014**, *6* (12), 6448-6451, 10.1039/C4NR01220H. DOI: 10.1039/C4NR01220H.

(24) Noh, J.; Yoon, C.-M.; Kim, Y. K.; Jang, J. High performance asymmetric supercapacitor twisted from carbon fiber/MnO2 and carbon fiber/MoO3. *Carbon* **2017**, *116*, 470-478. DOI: https://doi.org/10.1016/j.carbon.2017.02.033.
